# Crosstalk between mitochondrial homeostasis and AMPK pathway mediate the receptor-mediated cardioprotective effects of estradiol in ovariectomized female rats

**DOI:** 10.1371/journal.pone.0312397

**Published:** 2024-12-18

**Authors:** Mennatallah A. Gowayed, Zainab Zaki Zakaraya, Nehal Abu-Samra, Reem H. Elhamammy, Lobna M. Abdel Moneim, Hala A. Hafez, Ihab A. Moneam, Ghaleb A. Oriquat, Maher A. Kamel

**Affiliations:** 1 Department of Pharmacology &Therapeutics, Faculty of Pharmacy and Drug Manufacturing, Pharos University in Alexandria, Alexandria, Egypt; 2 Biopharmaceutics and Clinical Pharmacy Department, Faculty of Pharmacy, Al-Ahliyya Amman University, Amman, Jordan; 3 Department of Basic Sciences, Faculty of Physical Therapy, Pharos University in Alexandria, Alexandria, Egypt; 4 Department of Biochemistry, Faculty of Pharmacy, Alexandria University, Alexandria, Egypt; 5 Department of Biochemistry, Medical Research Institute, Alexandria University, Alexandria, Egypt; 6 Clinical Laboratory Sciences Department, College of Pharmacy, Almaaqal University, Basra, Iraq; 7 Supplementary General Sciences Department, Faculty of Dentistry, Future University, New Cairo, Egypt; 8 Department of Medical Laboratory Sciences, Faculty of Allied Medical Sciences, Al-Ahliyya Amman University, Amman, Jordan; Max Delbruck Centrum fur Molekulare Medizin Berlin Buch, GERMANY

## Abstract

Estrogen (E2) deficiency is a risk factor for cardiovascular disease (CVD), however, the exact mechanism for the E2 protective effect on CVD remains unclear. This study aimed to investigate the estrogen receptor (ER) and non-receptor mediated effects of E2 treatment on the cardiac expression of adenosine monophosphate-dependent protein kinase (AMPK), autophagic, mitophagy and mitochondrial homeostasis-regulating genes in ovariectomized (OVX) rats. Female rats were divided into two main groups; sham and bilaterally OVX rats, then each group was subdivided into four subgroups according to treatment; untreated, subcutaneously treated with E2 (30 μg/kg), or Fulvestrant (F) (5 mg/Kg), or a combination of both drugs for 28 days. The OVX rats or F-treated sham rats showed dyslipidemia, and marked disturbances in parameters of AMPK signaling, autophagy, mitophagy, mitochondrial fission, fusion and biogenesis. E2 administration to OVX or F-treated sham rats has corrected the disturbed lipid and cardiac profiles, increased AMPK, and restored the balance of cardiac autophagy, mitophagy, and mitochondrial dynamics and homeostasis. Most of these effects in OVX rats were blocked by the ER antagonist (F). Estrogen treatment has cardioprotective effects in OVX females through modulating cardiac mitochondrial homeostasis, mitophagy and autophagy and restoring the AMPK signaling pathway. As witnessed by Fulvestrant, these effects suggest the main role of ER-mediated signaling in regulating mitophagy and plasma and cardiac lipids along with the existence of a post-translational control mechanism or the involvement of estrogenic non-receptor pathway controlling the postmenopausal cardiac mitochondrial energy production machinery that needs further investigation.

## 1. Introduction

The incidence of cardiovascular disease (CVD) is associated with the decline in estrogen (E2) levels accompanied by menopause [1]. E2 through its receptors alpha (ERα) and beta (ERβ) can modulate the function of endothelial and vascular smooth muscle cells and vasodilators release such as nitric oxide, hence affecting the cardiovascular system [2].

Mitochondria constitute approximately 30% of the volume of cardiomyocytes and are essential for cardiac contractility and cell death. E2 can modulate mitochondrial function, homeostasis and dynamics exerting regulatory effects in numerous cardiovascular diseases. E2 can directly control essential genes for mitochondrial biogenesis, energy production, mitochondrial DNA (mtDNA) transcription, and survival [3]. It directly affects the nuclear respiratory factor 1 (NRF1) and peroxisome proliferator-activated receptor gamma coactivator 1 (PGC-1), two main key transcription factors that promote the transcription of other mitochondrial proteins essential for mitochondrial function [4].

Complementary to its role in mitochondrial biogenesis; E2/ERα signaling maintains mitochondrial homeostasis by regulating mitophagy, which selectively removes dysfunctional mitochondria in cardiac cells, mainly via modulating PTEN-induced kinase 1 (PINK1)-Parkin pathway, Beclin-1 and microtubule-associated protein 1 light chain 3 beta (LC3B). Any functional decline in mitophagy leads to the accumulation of dysfunctional mitochondria, a hallmark of many diseases [5]. It has also become evident that E2 regulates mitochondrial dynamics (fission/fusion); which play a critical role in maintaining functional mitochondria as well as being implicated in several cardiovascular diseases, by modifying proteins such as dynamin-related protein 1 (DRP1), and mitochondrial fusion protein 2 (MFN2) [6].

Mitochondrial damage or dysfunction is associated with defective folliculogenesis, impaired ovulation, and reproductive aging in females [7]. In contrast, E2 replacement provides beneficial effects on mitochondrial structure and function. By binding to its plasma membrane receptors, E2 initiates a phosphorylation cascade protecting the mitochondria from oxidative damage [8].

In addition, E2 regulates Adenosine monophosphate-dependent protein kinase (AMPK) in the cardiovascular system. AMPK also has antioxidant, anti-inflammatory, and metabolic benefits. Although the evidence for AMPK’s influence on the control of cardiometabolism is encouraging, little is known about how its dysregulation contributes to the emergence of cardiometabolic disorders [9].

Therefore, the current study aimed to evaluate the cardioprotective effects of estrogen in ovariectomized (OVX) rats and to explore the possible contribution of the cardiac expression of AMPK, autophagic and mitochondrial homeostasis regulating genes. Additionally, fulvestrant (F) (ICI 182,780), a selective estrogenic receptor downregulator (SERD), was used to investigate whether the receptor-mediated signaling was implicated or not.

## 2. Materials and methods

### 2.1 Experimental animals

Sixty-four female Wistar rats (200–220 g) were acquired from the animal facility of the Medical Research Institute, Alexandria University, Egypt. Animals were kept under observation for one week preceding the study with free access to food and water. Animal experiments were approved by the Institutional Animal Care and Use Committee of Alexandria University (AU01218123032), and comply with the guidelines of the National Institutes of Health for the care and use of Laboratory animals (NIH Publications No. 8023, revised 1978) and recommendations of Egypt’s guide for the care and use of laboratory animals [10]. The current study also adheres to the ARRIVE Guidelines for conducting *in vivo* experiments [11].

### 2.2 Experimental design

Rats were distributed into two groups at random. Group one (n = 32) was sham-operated and then allowed to recover for 4 weeks. It was then subdivided into subgroups (n = 8): group IA; the control group receiving the vehicle (corn oil), group IB; estradiol treated (E2, 30 μg/kg), group IC; fulvestrant treated (F, 5 mg/kg) [12] and group ID; treated with a combination of E2+F. Group two (n = 32) underwent a bilateral ovariectomization surgery (OVX); after being anesthetized, rats were bilaterally ovariectomized through a midline incision [13]. After 4 weeks of recovery, the OVX rats were divided into subgroups (n = 8): group IIA; untreated OVX group receiving the vehicle, group IIB receiving E2, group IIC receiving F, and group IID receiving E2+ F in the formerly stated doses. Drugs were subcutaneously injected at 09:00 a.m. for 28 days for 28 days.

### 2.3 Serum parameters

After the treatment duration, rats were euthanized (ketamine 100 mg/kg and xylazine 10 mg/kg) and blood was gathered by cardiac puncture to determine serum levels of atrial natriuretic peptide (Rat Atrial Natriuretic Peptide; ANP, cat. no. BYEK1390, Biospes, China), creatine kinase-MB (CK-MB) (cat. no. 239002, Spectrum, Egypt), and Troponin-I (rat cardiac troponin-I, cat no. CSB-E08594r, Cousabio, USA), analysis was performed according to manufacturer instructions.

Total cholesterol (TC) and triglyceride (TG) were measured colorimetrically (Boehringer Mannheim, Germany). The high-density lipoprotein cholesterol (HDL-c) was measured using the Lopes-Virella et al. method [14], magnesium chloride and phosphotungstic acid were used to precipitate an aliquot part of the serum, and then the cholesterol amount was measured in the supernatant. The low-density lipoprotein cholesterol (LDL-c) was then calculated using Friedewald´s equation [15]: LDL-c = TC–(HDL-c+ 1/5 TG).

### 2.4 Tissue parameters

Heart tissues were excised, washed with saline, and kept at -80°C till analysis. The excised cardiac tissues were divided into four aliquots: One aliquot was used for TG-myocyte amount determination using a modified Bligh and Dyer et al. method [16], and the second aliquot was used for total RNA extraction for quantitative real-time polymerase chain reaction (qPCR), the third aliquot used for total DNA extraction for assessment of mitochondrial DNA-copy number (mtDNA-CN) and the fourth aliquot homogenized in RIPA buffer pH 7.4 in the ratio of 1:9, then centrifuged at 10000 rpm, at 4° C for 20 minutes and the supernatants were stored at -20° C for subsequent determination of nitric oxide end-products (NOx) contents, AMPK, PGC-1α, PGC-1β, mitochondrial transcription factor A (TFAM), NRF1, Beclin-1 and LC3B protein contents.

### 2.5 Cardiac nitric oxide end-products (NOx)

Nitric oxide end-products (NOx) contents were analyzed following a simple Griess reaction [17]. The total protein in each sample was assessed using a modified Lowry *et al*. method [18].

### 2.6 ELISA determination

Measurement of AMPK (using Invitrogen™ AMPKα [pT172] ELISA Kit, cat. no. KHO0651, Austria), PGC-1α, PGC-1β, TFAM, NRF1, Beclin-1 and LC3B were analyzed using rat specific ELISA kits (cat. no. MBS2706379, MBS9718048, MBS1600609, MBS733192, MBS452829, and MBS1600540, MyBiosource, USA) according to manufacturer instructions.

### 2.7 Gene expression analysis using quantitative real-time polymerase chain reaction (qPCR)

The qPCR was used to quantify the cardiac expression of AMPK, PGC-1α, PGC-1β, TFAM, NRF1, Beclin-1, LC3B, DRP1, PINK1, and MFN2 in a two-step reverse transcriptase PCR. Total RNA was extracted utilizing the miRNeasy kit (Qiagen, Germany) following the manufacturer’s instructions. Then Reverse transcription was completed using miScript II RT Kit (Qiagen, Germany) upon the manufacturer’s instructions. The relative quantification was performed utilizing QuantiTect SYBR Green PCR Master Mix by Rotor-Gene Q qPCR. The quantitative PCR started with a preliminary denaturation (10 min at 95°C) and then amplification (45 cycles) as follows: Denaturation at 95°C for 5 seconds, annealing at 55°C for 15 seconds, and extension at 60°C for 15 seconds [19]. The used primers are obtainable in **Table 1**. The mRNA change in expression in each sample was quantified relative to the reference gene (18s rRNA) by normalizing the values of the threshold cycle (C_t_) of target mRNA using the 2^-ΔΔCt^ method [20].

**Table 1:** Primer sequences used for cDNA amplification.

Gene	Forward primer	Reverse primer	Accession No.
**AMPK**	CGCTTCCTGAACTTGTCC	GGTTGTCACCTGCTTCCA	NM_198769.2
**PGC-1α**	AGCTTTGGTCAGTTGGCT	CAGGATGACACCATTGAAGC	NM_019178.2
**PGC-1β**	TGATTAATGAATGAGTTCGGGC	TGCTCAGGAAACTTGACTGTTT	NM_012963.2
**TFAM**	CGCCTTATGTGGTGACTCGCTA	TCCTGGAAAGAGGATTTTGTGGC	NM_053829.2
**NRF1**	CCGAACGATACCAGAACCTGTC	ACGCAACTCTCGGTAGGTCCTT	NM_031020.3
**Beclin-1**	GGCTTTCTGACGGAGTATGTGG	GTTGGAGAGCATCTCAGCCAGA	NM_017347.3
**LC3B**	CAGGATCCATGCCGTCCCAGAAGACC	GTCCCTTTTTGCCTTGGTAG	NM_022867.2
**DRP1**	GATGCCATAGTTGAAGTGGTGAC	CCACAAGCATCAGCAAAGTCTGG	NM_053655.3
**PINK1**	GGTGTCAGGCTGGGGCAA	TGGCTTCATACACAGCGGC	NM_001106694.1
**MFN2**	GCCAGCTTCCTTGAAGACAC	GCAGAACTTTGTCCCAGAGC	NM_130894.4
**18S rRNA**	GTAACCCGTTGAACCCCATT	CAAGCTTATGACCCGCACTT	NR_046237.2

### 2.8 The mitochondrial DNA-copy number (mtDNA-CN)

The mtDNA, relative to nuclear DNA, was assayed using real-time PCR [21]. After total genomic DNA isolation using DNeasy kit (Qiagen, Germany), we used a specific primer pair for mtDNA sequence (NC_040919.1, Forward; AATGGTTCGTTTGTTCAACGATT and Reverse; AGAAACCGACCTGGATTGCTC) and a primer pair specific for the nuclear PGC-1α gene (NM_031347.1, Forward; ATGAATGCAGCGGTCTTAGC, and Reverse; AACAATGGCAGGGTTTGTTC) to perform PCR cycles under the following conditions: 98˚C for 2 min followed by 45 cycles of 98˚C for 10 sec and 55˚C for 30 sec. The relative mtDNA signal to the nuclear DNA signal was calculated. The nuclear gene was used to quantify nuclear DNA (nDNA) and, therefore, normalization of the mtDNA amount per the nDNA of the cells using the equation: R = 2^-ΔCt^ where ΔCt = Ct_mtDNA_–Ct_nuclear_.

### 2.9 Statistical analysis

The data are expressed as mean ± SD (n = 8). Data were analyzed using SPSS software package version 18.0 (SPSS, Chicago, IL, USA). Multiple comparisons between different groups were analyzed using one-way analysis of variance (ANOVA), followed by Tukey multiple comparison post hoc test. The differences were considered significant at *p*<0.05.

## 3. Results

### 3.1 Serum lipid profile and TG-myocyte

As presented in Table 2, The OVX untreated rats showed a significant increase in the lipid profile parameters and TG-myocytes except HDL-c decreased compared to the sham vehicle. Administration of F to sham or OVX rats significantly increased serum TG, cholesterol, LDL-c and TG-myocytes and decreased HDL-c compared to their corresponding vehicles. The effect of F on sham rats was comparable to the OVX vehicle. Upon E2 administration to OVX rats, the lipid profile parameters and TG-myocytes returned to normal except for serum TG which decreased but was still elevated compared to the sham vehicle. The E2 effect on OVX rats was significantly higher in serum TG and cholesterol compared to E2-treated sham rats. In contrast, the E2 administration alone or in combination with F to sham rats showed no significant difference compared to the sham vehicle. The combination of F with E2 in OVX rats suppressed the effect of E2 as it showed no significant difference in lipid profile parameters and TG-myocytes compared to F alone and OVX vehicle.

**Table 2:** Serum lipid profile and TG-myocytes in the different studied groups.

		TG (mg/dl)	Cholesterol (mg/dl)	HDL–c (mg/dl)	LDL–c (mg/dl)	TG- Myocytes (mg/g tissue)
Sham	Vehicle	49.4 ± 6.7	116 ± 8.73	45.6 ± 1.14	60.3 ± 9.06	28.4 ± 3.56
	E2	48.4 ± 9.2	109^a^ ± 11.4	47.8 ± 2.59	51.3 ± 8.1	31.6 ± 2.30
	F	125^ab^ ± 9.2	157^ab^ ± 19.2	39.2^b^ ± 3.8	93.2^b^ ± 18.4	44.6^ab^ ± 2.88
	E2+ F	61.4^c^ ± 10.4	137^b^ ± 12.5	45.8 ± 2.05	78.7^b^ ± 12.6	34.8^c^ ± 5.02
OVX	Vehicle	138^abd^ ± 7.4	158^ab^ ± 10.6	38.5^b^ ± 5.09	91.6^b^ ± 15.1	50.4^abd^ ± 4.39
	E2	85.1^abcd^ ± 6.4	127^b^ ± 15.8	46.4^ce^ ± 3.4	65.6 ± 15.3	36.8^ce^ ± 4.63
	F	145^abdf^ ± 12.5	166^abf^ ± 12.6	36.4^abdf^ ± 3.68	100^abf^ ± 13.2	55.8^abcdf^ ± 7.85
	E2+ F	124^abdf^ ± 19.7	162^abf^ ± 16.7	38.8^bf^ ± 3.35	98.7^abf^ ± 21.3	46.8^abdf^ ± 2.77

Values are presented as means ± SD, n = 8 (^a^ significant difference compared with Sham-vehicle, ^b^ significant difference compared with Sham-E2, ^c^ significant difference compared with Sham-F, ^d^ significant difference compared with Sham-E2+F, ^e^ significant difference compared with OVX-Vehicle, ^f^ significant difference compared with OVX-E2, and ^g^ significant difference compared with OVX-E2+F) using the one-way ANOVA Test followed by the Tukey post hoc test at p> 0.05. E2; estradiol, F; fulvestrant, OVX; ovariectomized rats.TG; triglyceride, HDL-c; high-density lipoprotein cholesterol, LDL-c; low-density lipoprotein cholesterol.

### 3.2 Serum cardiac profile and cardiac content of NOx

In sham rats, the F-sham treated rats show a significant elevation in serum CK-MB and Troponin-I and a significant decline in NOx compared with the sham vehicle rats, as shown in Table 3. The co-administration of E2 with F significantly reversed the effects of F on the sham females partially on CK-MB and completely on troponin-I and NOx levels. In OVX rats, the untreated rats showed a significant increase in CK-MB and troponin-I and a significant decrease in cardiac NOx compared to the sham vehicle. The F-treatment of OVX rats significantly worsened the increase in cardiac profile parameters and the decline in cardiac NOx and ANP compared to the OVX vehicle rats. E2 treatment of OVX rats significantly normalized ANP, Troponin-I, and NOx, and significantly declined the CK-MB compared with the OVX vehicle group. Co-administration of F with E2 in OVX rats significantly blocked the ameliorative effects of E2 on CK-MB and NOx levels but had no significant effects on ANP and troponin-I levels (Table 3).

**Table 3:** Serum cardiac profile and cardiac content of NOx in the different studied groups.

		ANP (ng/ml)	CK-MB (U/l)	Troponin-I (ng/ml)	NOx (μmol/mg)
Sham	Vehicle	0.330 ±0.051	12.4 ± 1.10	0.170 ± 0.015	45.5 ± 3.56
	E2	0.344 ± 0.029	14.3 ± 1.62	0.167 ± 0.024	46.4 ± 3.04
	F	0.271 ± 0.036	20.7^ab^ ± 1.92	0.233^ab^ ± 0.019	32.9^ab^ ± 2.96
	E2+ F	0.342 ± 0.044	16.4 ± 2.15	0.186^c^ ± 0.025	43.8^c^ ± 4.59
OVX	Vehicle	0.263 ± 0.029	27.3^abcd^ ± 2.33	0.254^abd^± 0.024	22.4^abcd^ ± 1.65
	E2	0.332 ± 0.038	18.8^ae^ ± 1.66	0.216 ± 0.014	40.7^ce^ ± 3.03
	F	0.259^b^ ± 0.044	30.9^abcdf^ ± 3.81	0.263^abdf^ ±0.026	23.4^abcdf^ ± 2.04
	E2+ F	0.290± 0.052	23.7^abdefg^ ± 2.40	0.238^abd^ ±0.020	34.3^abdeg^ ± 3.13

Values are presented as means ± SD, n = 8 (^a^ significant difference compared with Sham-vehicle, ^b^ significant difference compared with Sham-E2, ^c^ significant difference compared with Sham-F, ^d^ significant difference compared with Sham-E2+F, ^e^ significant difference compared with OVX-Vehicle, ^f^ significant difference compared with OVX-E2, and ^g^ significant difference compared with OVX-E2+F) using the one-way ANOVA Test followed by the Tukey post hoc test at p> 0.05. E2; estradiol, F; fulvestrant. OVX; ovariectomized rats. ANP; Atrial natriuretic peptide, CK-MB; creatine kinase-MB. NOx; nitric oxide.

### 3.3 Cardiac autophagy parameters

**Fig 1:**
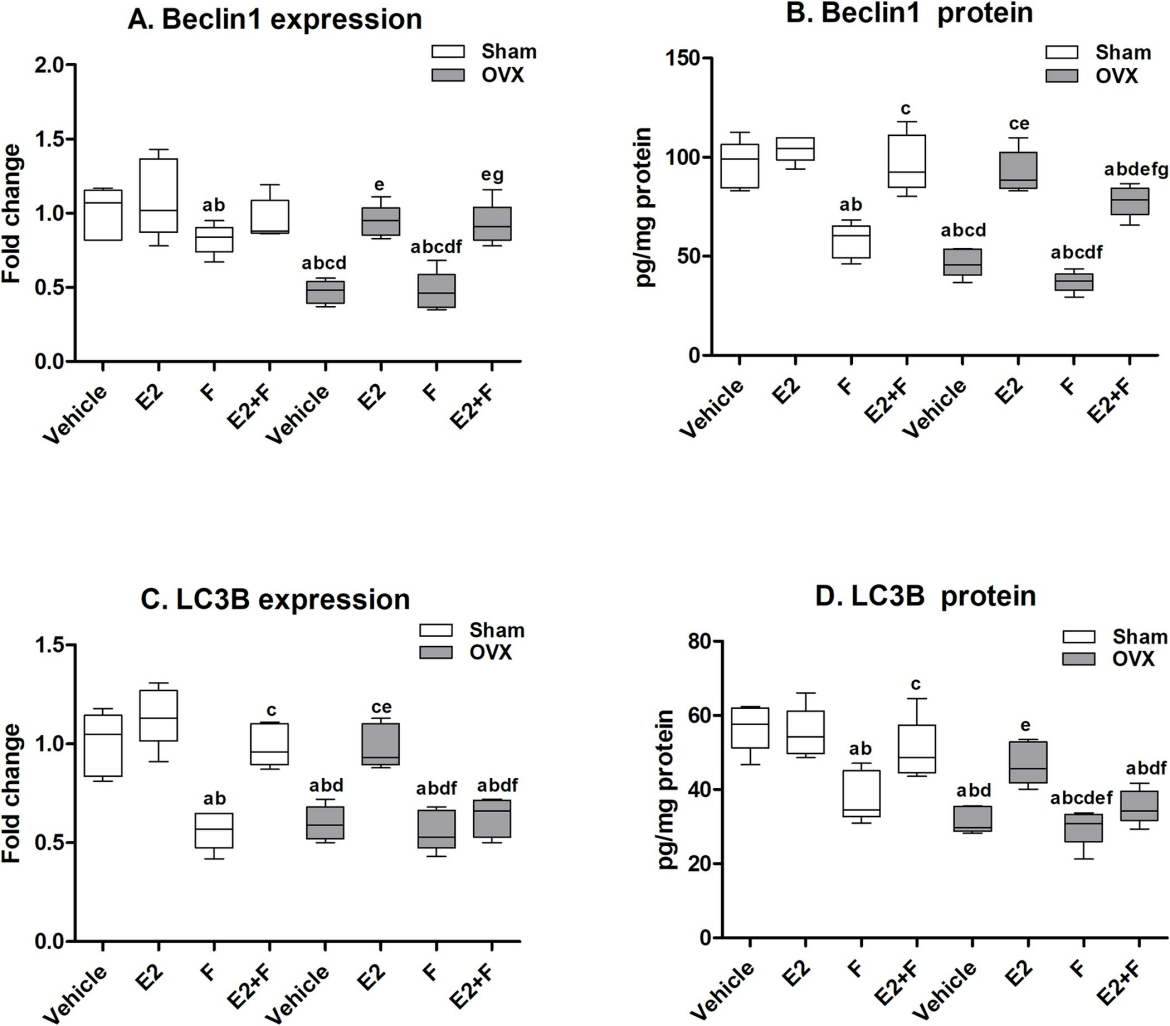
Cardiac autophagy parameters. (A-B) Beclin-1, (C-D) microtubule-associated protein 1 light chain 3 beta (LC3B). E2; estradiol, F; fulvestrant, OVX; ovariectomized rats. Values are presented as means ± SD (n = 8) (^a^ significant difference compared with Sham-vehicle, ^b^ significant difference compared with Sham-E2, ^c^ significant difference compared with Sham-F, ^d^ significant difference compared with Sham-E2+F, ^e^ significant difference compared with OVX-Vehicle, ^f^ significant difference compared with OVX-E2, and ^g^ significant difference compared with OVX-E2+F) using the one-way ANOVA Test followed by the Tukey post hoc test at p> 0.05.

In sham rats, F administration showed a significant decrease in the Beclin-1 and LC3B mRNA expressions and protein levels compared to sham vehicle and E2-treated sham, as presented in Fig 1. The co-administration of E2 with F to sham rats significantly reversed F effects on the Beclin-1 and LC3B protein levels and LC3B mRNA expressions. The OVX untreated rats showed a significant decline in Beclin-1 and LC3B mRNA expressions and protein levels compared to the sham vehicle. F-treated OVX rats showed a significant decrease in Beclin-1 and LC3B mRNA expressions and protein levels compared to E2-treated OVX, a similar effect to that of the OVX vehicle. The effect of F on sham rats was comparable to that of the OVX vehicle rats in LC3B mRNA expression and protein level, while Beclin-1 mRNA expression and protein level were significantly higher than the OVX vehicle rats. E2-treated OVX showed a significant increase in Beclin-1 and LC3B mRNA expressions and protein levels compared to the untreated OVX vehicle and completely normalized Beclin-1 and LC3B mRNA expressions and protein levels compared to the sham vehicle and E2-treated sham. The combination of E2+F to OVX rats showed a significant partial reversal of F effects on Beclin-1 protein level while F did not block E2 effects on Beclin-1 mRNA expression. On the other hand, the co-administration of F with E2 to OVX rats significantly blocked the effect of E2 on LC3B mRNA expression and protein level as there was no significant difference with the OVX vehicle and F-treated OVX (Fig 1).

### 3.4 Cardiac mitophagy parameters

**Fig 2:**
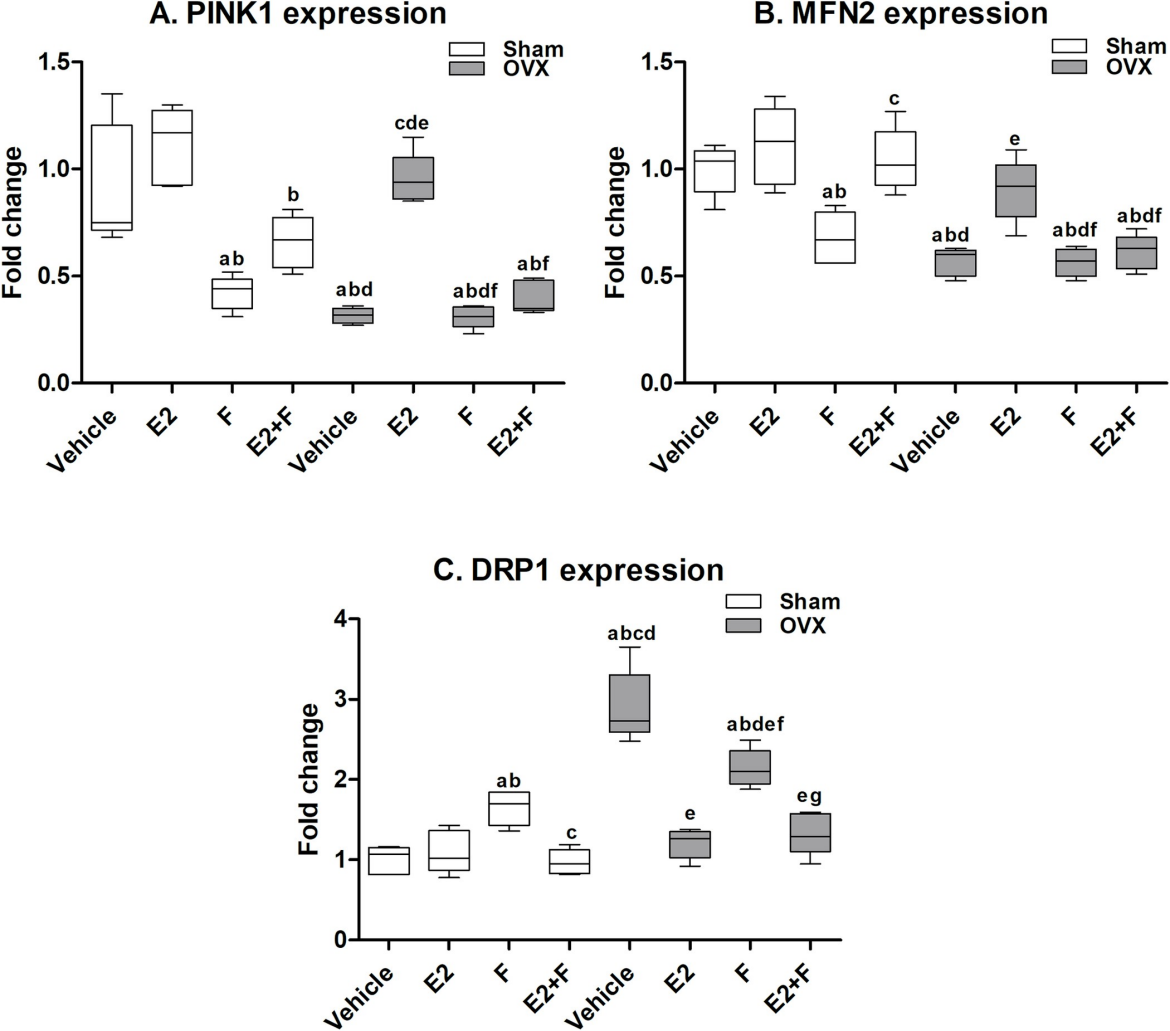
Cardiac mitophagy parameters. (A) PTEN-induced putative kinase 1 (PINK1), (B) mitochondrial fusion protein 2 (MFN2), and (C) dynamin-related protein 1 (DRP1). E2; estradiol, F; fulvestrant, OVX; ovariectomized rats. Values are presented as means ± SD (n = 8) (^a^ significant difference compared with Sham-vehicle, ^b^ significant difference compared with Sham-E2, ^c^ significant difference compared with Sham-F, ^d^ significant difference compared with Sham-E2+F, ^e^ significant difference compared with OVX-Vehicle, ^f^ significant difference compared with OVX-E2, and ^g^ significant difference compared with OVX-E2+F) using the one-way ANOVA Test followed by the Tukey post hoc test at p> 0.05.

In sham rats, the F-treated sham showed significant downregulation in PINK1 and MFN2 mRNA expression and a significant upregulation in DRP1 mRNA expression compared to the sham vehicle, as presented in Fig 2. The co-administration of E2 with F to sham rats significantly reversed the F effect on MFN2 and DRP1 mRNA expressions and did not reverse PINK1 expression compared to F-treated and E2-treated sham. The OVX untreated rats showed a significant decrease in PINK1 and MFN2 mRNA expression and a significant increase in DRP1 mRNA expression compared to the sham vehicle. The F administration to OVX rats showed no significant difference in the PINK1 and MFN2 mRNA expressions and significantly decreased DRP1 mRNA expression compared to the OVX vehicle. Upon E2 administration to OVX rats, the mRNA expressions of PINK1 and MFN2 showed a significant increase while DRP1 mRNA expression showed a significant decrease compared to OVX vehicle. The co-administration of F with E2 to OVX rats significantly blocked the effect of E2 on the PINK1 and MFN2 mRNA expressions while not blocking the E2 effect on DRP1 mRNA expression (Fig 2).

### 3.5 Cardiac mitochondrial biogenesis parameters

#### 3.5.1 Cardiac AMPK-related mitochondrial biogenesis

**Fig 3:**
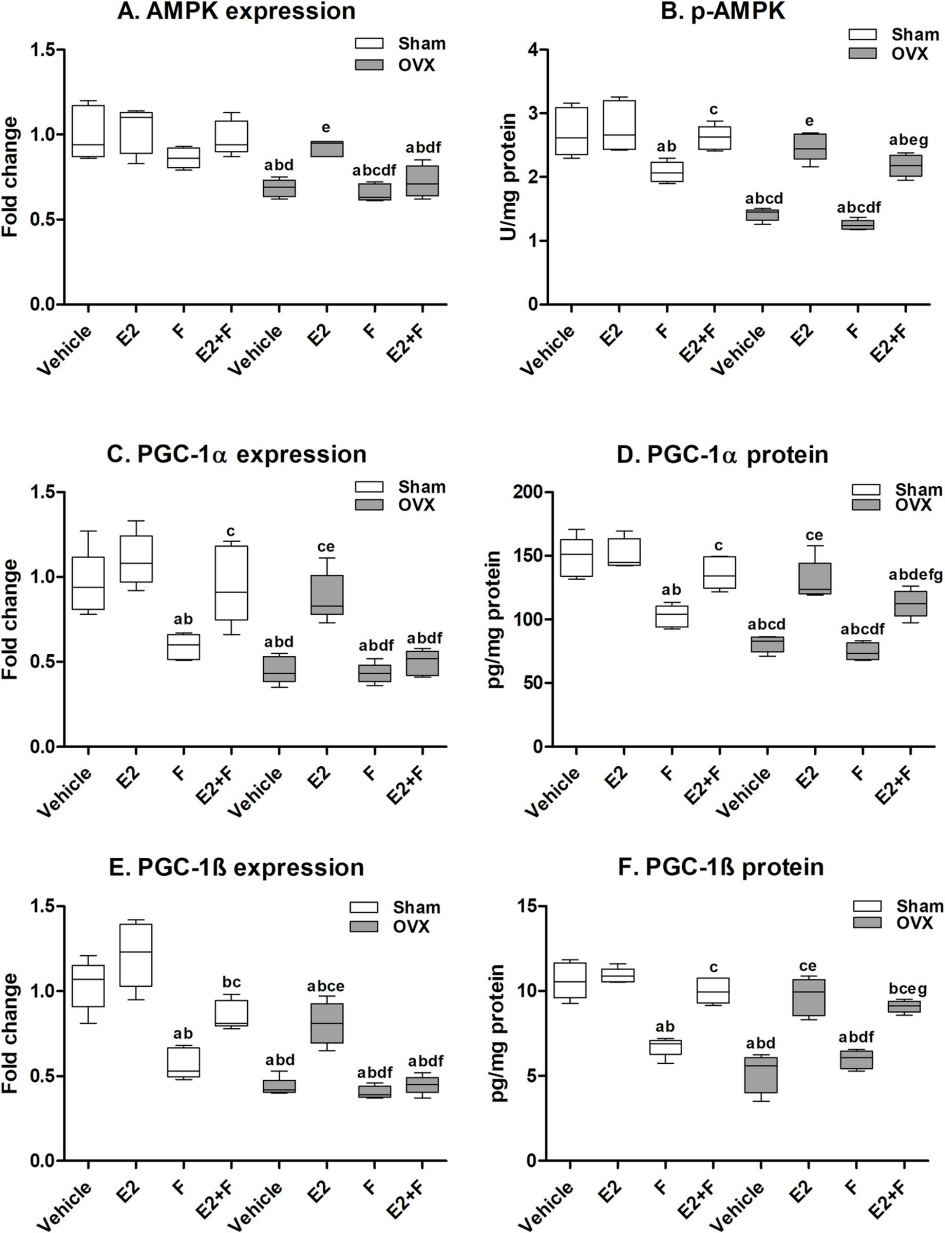
Cardiac AMPK-related mitochondrial biogenesis. (A-B) AMPK, (C-D) peroxisome proliferator-activated receptor gamma coactivator 1-alpha (PGC-1α) and (E-F) peroxisome proliferator-activated receptor gamma coactivator 1- beta (PGC-1β). E2; estradiol, F; fulvestrant, OVX; ovariectomized rats. Values are presented as means ± SD (n = 8) (^a^ significant difference compared with Sham-vehicle, ^b^ significant difference compared with Sham-E2, ^c^ significant difference compared with Sham-F, ^d^ significant difference compared with Sham-E2+F, ^e^ significant difference compared with OVX-Vehicle, ^f^ significant difference compared with OVX-E2, and ^g^ significant difference compared with OVX-E2+F) using the one-way ANOVA Test followed by the Tukey post hoc test at p> 0.05.

As presented in Fig 3, the F-treated sham showed a significant reduction in p-AMPK protein level, PGC-1α and PGC-1β mRNA expression and protein level compared to the sham vehicle and E2-treated sham. The combination of E2 and F in sham rats reversed the F effects on AMPK, PGC-1α, PGC-1β protein levels, and PGC-1α mRNA expression while partial reversal of the mRNA expression of AMPK and PGC-1β was observed. The OVX untreated rats showed significantly reduced mRNA expression and protein level of AMPK, PGC-1α and PGC-1β compared to the sham vehicle. F-treated OVX showed a significant decrease in the mRNA expression and protein level of AMPK, PGC-1α and PGC-1β compared to E2-treated OVX, an effect that was comparable to that of the OVX vehicle. The effect of F administration on sham rats was comparable to that of the OVX vehicle on AMPK, PGC-1α mRNA expressions and PGC-1β mRNA expression and protein level. E2 administration to OVX rats significantly normalized the AMPK, PGC-1α mRNA expression and protein level and PGC-1β protein level compared to sham vehicle, E2-treated sham and OVX vehicle. The mRNA expression of PGC-1β in the E2-treated OVX significantly increased compared to the OVX vehicle but still lower than the sham vehicle and E2-treated sham. The co-administration of F with E2 to OVX rats significantly blocked the effect of E2 on the mRNA expression of AMPK, PGC-1α and PGC-1β. On the other hand, F did not block the E2 effects at the protein levels of AMPK and PGC-1β in E2+F OVX while partially blocking the protein level of PGC-1α (Fig 3).

#### 3.5.2 Cardiac mitochondrial DNA-related mitochondrial biogenesis

**Fig 4:**
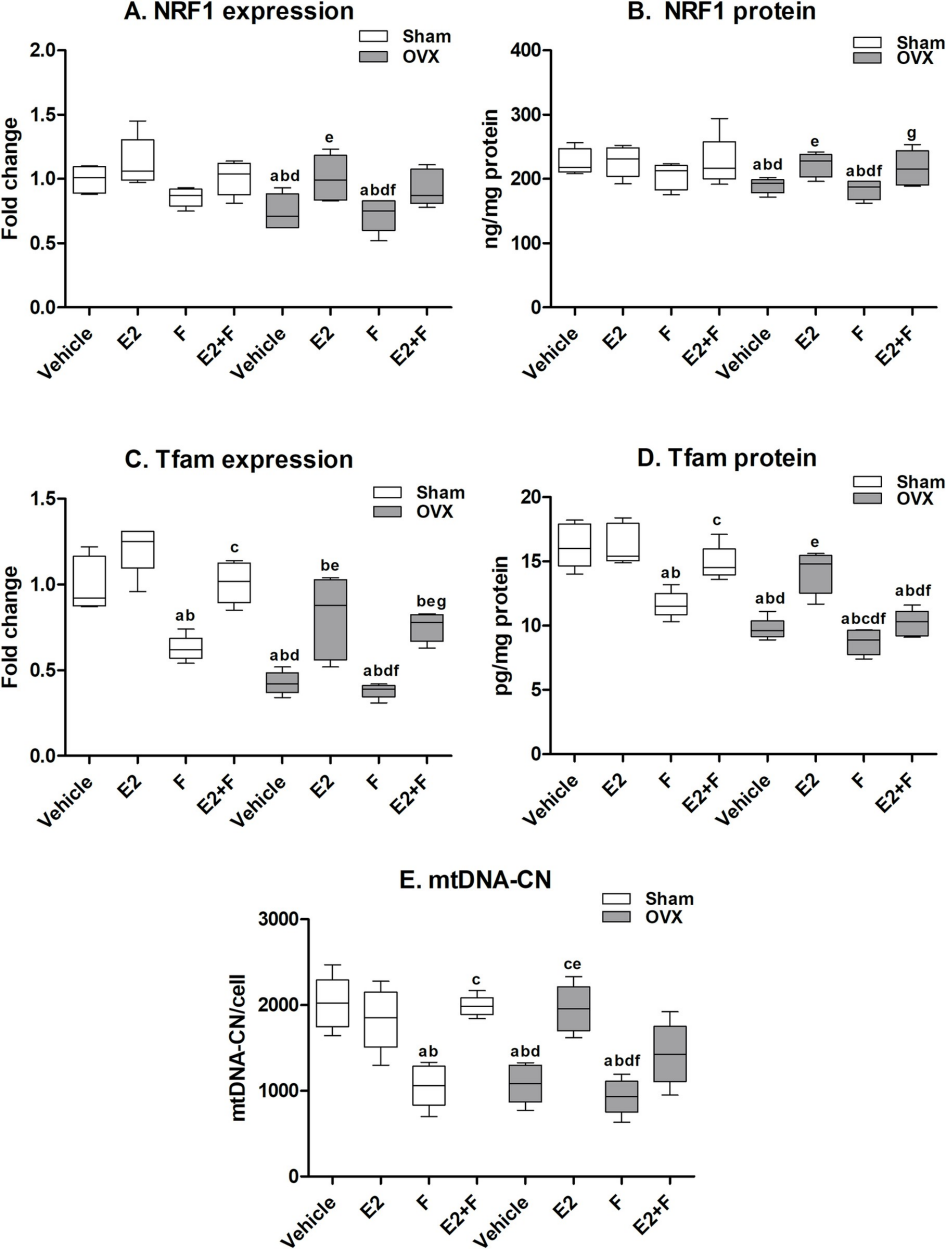
Cardiac mitochondrial biogenesis parameters. (A-B) nuclear respiratory factor 1 (NRF1), (C-D) mitochondrial transcription factor A (TFAM), and (E) mitochondrial DNA copy number (mtDNA-CN). E2; estradiol, F; fulvestrant, OVX; ovariectomized rats. Values are presented as means ± SD (n = 8) (^a^ significant difference compared with Sham-vehicle, ^b^ significant difference compared with Sham-E2, ^c^ significant difference compared with Sham-F, ^d^ significant difference compared with Sham-E2+F, ^e^ significant difference compared with OVX-Vehicle, ^f^ significant difference compared with OVX-E2, and ^g^ significant difference compared with OVX-E2+F) using the one-way ANOVA Test followed by the Tukey post hoc test at p> 0.05.

As presented in Fig 4, F administration to sham rats showed no significant decrease in the NRF1 mRNA expression and protein level compared to the sham vehicle and E2-treated sham. The F-treated sham showed a significant reduction in TFAM mRNA and protein level, and mtDNA-CN compared to the sham vehicle and E2-treated sham. The combination of E2 with F in sham rats showed that E2 reversed F effects on TFAM at mRNA and protein levels and mtDNA-CN. The OVX untreated rats showed significantly reduced NRF1, TFAM mRNA expressions and protein levels and mtDNA-CN compared to the sham vehicle. F administration to OVX rats showed a significant reduction in these parameters compared to E2-treated OVX, an effect comparable to the OVX vehicle. The effect of F on sham rats was comparable to that of the OVX vehicle in NRF1, TFAM mRNA expressions and protein levels and mtDNA-CN. The E2-treated OVX showed a significant increase in the mRNA expressions and protein levels of NRF1, TFAM and mtDNA-CN compared to the OVX vehicle. The co-administration of F with E2 to OVX rats partially blocked the E2 effects on the NRF1 mRNA expression and mtDNA-CN. The administration of E2+F to OVX rats significantly blocked the E2 effects on TFAM protein levels however, it did not block the TFAM mRNA expressions and NRF1 protein level (Fig 4).

### 3.6 Correlations study

The correlations data in OVX groups of rats indicated that the cardiac AMP expression and its active phosphorylated form are positively correlated with mtDNA-CN, proteins regulating mitochondrial biogenesis (PGC-1α, PGC-1β, NRF1, and TFAM), autophagy and mitophagy markers (Beclin-1, LC3B, and PINK1), and mitochondrial fusion marker (MFN2), while negatively correlated with mitochondrial fission marker (DRP-1). Also, mtDNA-CN is positively correlated with PGC-1α, PGC-1β, NRF1, TFAM, Beclin-1, LC3B, and PINK1 and negatively correlated with DRP-1. Cardiac protein contents of both PGC-1α, and PGC-1β are positively correlated with NRF1, TFAM, Beclin-1, LC3B, and PINK1 and negatively correlated with DRP-1 (Table 4).

**Table 4:** Correlation coefficient between different markers in the OVX rats.

	mtDNA-CN	PGC-1α Protein	PGC-1β Protein	NRF1 Protein	TFAM Protein	Beclin-1 Protein	LC3B Protein	PINK1 mRNA	MFN2 mRNA	DRP-1 mRNA
**AMPK mRNA**	.660**	.704**	.660**	.381	.891**	.748**	.681**	.813**	.762**	-.598**
**p-AMPK Protein**		.892**	.875**	.688**	.763**	.919**	.724**	.779**	.712**	-.771**
**mtDNA-CN**		.794**	.591**	.653**	.746**	.803**	.798**	.815**	.672**	-.538*
**PGC-1α Protein**			.861**	.596**	.668**	.924**	.716**	.845**	.678**	-.699**
**PGC-1β Protein**				.586**	.600**	.867**	.602**	.652**	.647**	-.857**

**. Correlation is significant at the 0.01 level (2-tailed).

*. Correlation is significant at the 0.05 level (2-tailed).

## 4. Discussion

The findings of the present study indicated that the ovariectomy-associated E2 deficiency and the impaired E2-action using ER antagonist (Fulvestrant; F) disturbed the lipid profile, caused ectopic accumulation of triglycerides in myocytes, elevated serum cardiac profile parameters, disturbed cardiac nitric oxide production, and autophagy markers, deregulated mitochondrial homeostasis and biogenesis and impaired AMPK pathway. Upon E2 administration, the distortion in those cardiac parameters was completely ameliorated in sham rats while in OVX rats, E2 supplementation corrected most of the parameters. The co-supplementation of OVX rats with E2 and F indicated that F completely blocked the ameliorative effects of E2 on lipid profile, mitochondrial biogenesis, fusion, mitophagy and AMPK pathway while only partially blocking its effects on serum cardiac profile, autophagy and mitochondrial fission which may imply that the cardioprotective actions of E2 in the OVX rats are modulated mainly through the receptor-mediated mechanism while other actions are mediated through the non-ER pathways.

In line with previous studies, the disturbed serum lipid profile and TG-myocytes in the current study indicate a possible risk of building up fats and cholesterol in the arteries and hence contributing to possible cardiac complications [22, 23]. Following earlier studies [24–26], E2 replacement therapy reversed the disturbed lipid profile in OVX rats, showing decreased serum and TG-myocyte levels, serum cholesterol and LDL-cholesterol while increasing HDL-cholesterol levels. In the current study, the combination of F with E2 in OVX rats suppressed the effect of E2 as it showed no significant difference in lipid profile parameters and TG-myocytes compared to F alone and OVX vehicle. This finding confirms the role of E2 in protecting the cardiomyocytes and this protection could be through an ER-mediated pathway. Such effect is attributed to the vasodilatory effect of E2 on the endothelium. A previous study by Koh et al. [27] noticed that combining E2 with the cholesterol-lowering therapeutic plan of postmenopausal women shows unique properties on the vasculature and remarkably improves markers of vascular inflammation and fibrinolysis.

Knowing that E2 has a rapid vasodilatory effect mediated by NO production and a long-term effect on the vasculature by regulation of vascular gene expression [28], measuring NOx levels in the heart tissues of E2-deficient OVX and F-treated rats has shown a significant decrease compared to sham rats. In line with the current study, Maleki et al. [25] reported that E2 increased the NO levels and endothelial nitric oxide synthases (eNOS) that were decreased in OVX rats. Although ERs may regulate NO production, the E2 and F combination, in the current study, in OVX rats significantly increased NOx levels over OVX vehicle and F-treated OVX. This finding suggests that the effect of E2 on NOx may also be regulated through other non-ER-mediated signaling, like the direct activation of the endothelial nitric oxide synthase (eNOS) by E2 [29, 30].

Regarding cardiac profile, ANP is a cardiac hormone that stimulates vasodilation and is used as a marker of myocyte hypertrophy [31]. In the present study, serum ANP in the OVX rats showed no significant decrease compared to sham rats after 8 weeks of recovery and treatment. This was in line with a previous study in which the level of ANP was unchanged after 3 and 6 weeks of OVX rats compared to sham [32]. Serum levels of CK-MB and troponin-I, the markers of myocyte necrosis [33], indicate an affected heart tissue upon E2-deficiency in OVX and F-treated rats. The co-administration of E2 with F in OVX rats showed a mild decline in CK-MB and Troponin-I levels compared to the OVX vehicle and F-treated OVX and a mild increase compared to E2-treated OVX, these effects suggest that E2 may regulate CK-MB and troponin-I through ER-mediated and non-ER-mediated signaling. One of those non-ER signaling might be related to serum cortisol levels, knowing that serum cortisol levels are affected by E2 and directly relate to serum CK-MB and troponin-I levels [34–36].

Regarding metabolic homeostasis, the OVX-vehicle and the F-treated sham groups in this study showed a reduction in the cardiac expression of AMPK and its active phosphorylated form, where the aberration of both was reversed by E2 administration. This aligns with other findings reporting that ER activation in OVX wild-type rats activates AMPK signaling [37]. It is to be noted that F in OVX has completely blocked the action of E2 on AMPK expression, but only partially suppressed its phosphorylation suggesting the presence of a post-translational control mechanism or the involvement of an alternate estrogenic pathway other than ER in regulating AMPK activation by phosphorylation.

AMPK is a master metabolic regulator that controls multiple metabolic processes in response to outside and inside stressors. Dysregulation of AMPK activity and downstream targets is implicated in many diseases, including metabolic diseases, aging, cancer, cardiovascular diseases, neurological diseases, inflammation, and immunity [38, 39]. The heart is an organ with high energy demands, relying on an abundant and constant energy supply to exert its normal function. Mitochondria is the main player in providing this energy production by oxidative phosphorylation. So, the mitochondrial homeostasis and quality in cardiomyocytes must be strictly maintained and the damaged or superfluous mitochondria are removed by mitophagy. AMPK activity is implicated in the regulation of these different processes [38–40]. The central role of AMPK in the regulation of cardiac mitochondrial homeostasis and biogenesis is confirmed in the present study by the correlation data which indicated that AMP expression and its active phosphorylated form are positively correlated with mtDNA copy number, proteins regulating mitochondrial biogenesis (PGC-1α, PGC-1β, NRF1, and TFAM), autophagic markers (Beclin-1 and LC3B), mitophagy marker (PINK1), and mitochondrial fusion marker (MFN2), while negatively correlated with mitochondrial fission marker (DRP-1).

It is well established that E2 deficiency whether age-associated or induced leads to cardiac mitochondrial dysfunction [3, 41]. In line with the current study, Zhao et al. [42] reported that mitochondrial function, biogenesis and dynamics were impaired due to OVX-induced E2 deficiency. Other studies also confirmed the findings of the current study in which OVX disrupted cardiac mitochondrial biogenesis whereas E2 counteracted these effects [43, 44]. By binding to ERα and ERβ, E2 activates some transcription factors that control genomic and mtDNA, including the activation of AMPK that stimulates PGC1α/β. In turn, some coactivators like NRF1 and TFAM will interact to regulate mtDNA and enhance mitochondrial biogenesis and survival in menopause [3, 45]. These reports were in line with the present correlation data which confirm the positive correlation between the cardiac phosphorylated AMPK with PGC-1α, PGC-1β, NRF1, and TFAM and the positive association between these proteins with the cardiac mtDNA copy number. The co-administration of F with E2 indicated different patterns of ER blockage on most of the cardiac mitochondrial biogenesis pathway especially between the mRNA and protein levels, suggesting the presence of a post-translational control mechanism or the involvement of an alternate estrogenic pathway other than ER in controlling the postmenopausal cardiac mitochondrial energy production machinery.

Decreased mtDNA-CN indicates a higher risk of CVD [46]. The OVX rats and F-treated sham showed a significant decline in the mtDNA-CN indicating mitochondrial dysfunction and a greater risk for CVD. E2 administration exerted a cardioprotective action as it increased the mtDNA-CN in OVX. In line with the current study, Galmés-Pascual et al. [44] reported that mtDNA-CN increased upon E2 replacement therapy compared to OVX rats. The co-administration of F with E2 in OVX showed partial blockage of the E2 effect on mtDNA-CN suggesting the existence of another regulatory estrogenic pathway than the classical ER-mediated signaling.

In addition to its role in cardiac mitochondrial biogenesis, AMPK has an additional role in regulating autophagy and mitophagy as well as mitochondrial dynamics [5, 38]. Activation of AMPK exerts a cardiac protective effect which is largely dependent on the inhibition of DRP1-mediated mitochondrial fission while promoting mitochondrial fusion [47]. This is consistent with the findings of the present study as evidenced by the positive correlation between AMPK expression and phosphorylated protein form and MFN2, while negatively correlated with DRP-1

Interestingly, F did not block the effect of E2 on DRP1, exhibiting a similar pattern to p-AMPK, again illustrating that mitochondrial fission in the heart via the AMPK-DRP1 axis is not solely under estrogenic control by ERα. In contrast, F completely blocked the effect of E2 on MFN2 expression which illustrates the potential role of E2 regulating mitochondrial fusion and MFN2 expression via ERα, in addition to ERβ, as previously reported in MCF7 cell line, as well as GPER with its role in stimulating fusion [48]. This raises the possibility that F can downregulate ERα as well as GPER [3].

In the same context, mitophagy is essential for maintaining mitochondrial homeostasis. The most studied mechanism for mitophagy is via the PINK1-Parkin pathway, and evidence from cardiovascular models highlights the role of AMPK in regulating PINK1-Parkin-dependent mitophagy [38]. Beclin-1 is a key component of the autophagy machinery in the heart. The results of the present study show again that E2-mediated activation of AMPK was paralleled with the upregulation of mitophagy and autophagic markers including PINK1-LC3B and Beclin-1 with subsequent cardiac protection, in agreement with evidence that cardiac myopathies can be ameliorated by enhancing autophagy [49].

Fulvestrant was able to completely block the effect of E2 on PINK1-LC3B expression and phosphorylation to reinforce that the estrogenic control of the main route for mitophagy and autophagy via PINK1 and LC3B in the heart is under the control of ER signaling. It is to be noted that, fulvestrant did not block the E2-induced upregulation of Beclin-1 expression, but only partially blocked its protein level which implicates ER involvement in the post-translational modification of Beclin-1 in cardiac autophagy.

It is noteworthy that this study showed that the cardiac protective effect of E2 was in part due to promoting autophagy, mitophagy and mitochondrial fusion while suppressing mitochondrial fission, although there is enough evidence supporting the necessity of mitochondrial fission for mitophagy to occur. However, sometimes mitophagy can occur independent of fission and the mitochondrial fragmentation mediated by mitochondrial fission may not be a prerequisite for mitophagy [5].

Moreover, our data agree with the reported balance existing between mitochondrial fusion and fission and how they counterbalance each other to control the structure of the mitochondria [5]. Excessive mitochondrial fission can be detrimental, and it is clear that fission was significantly upregulated in OVX compared to the control group, being a pathological mechanism in cardiac dysfunction suppressed by E2 supplementation [47]. In contrast, mitophagy as a protective mechanism was significantly reduced in OVX and enhanced by E2 to eliminate dysfunctional mitochondria and restore cardiac function. Therefore, fission could be either positively or negatively correlated with mitophagy in a context-dependent manner in the heart.

Taking into consideration the differential effects of fulvestrant on the sham and the OVX rats in the current study it significantly affected almost all parameters in the sham rats, while it had no significant effects in the OVX rats. The explanation may be a result of the pure antiestrogenic nature of the drug which depends on blocking the action of E2 through antagonizing the activation of ER. So, its main effects depend on the presence of the physiological levels of E2 [50]. In sham rats, normal physiological E2 produces its effects through its action in ER. The treatment with Fulvestrant inhibited these effects, induced the significant changes observed in most studied parameters, and caused what may be called chemically induced ovariectomy. While the OVX rats were estrogen-deficient and already lacked E2 actions, the treatment with Fulvestrant didn’t expect to induce further deterioration compared with the OVX rats. Besides the fact that the antagonist activity of fulvestrant and its ability to induce ERα degradation are not coupled processes [51], elucidates an unclear mechanism of action of fulvestrant that still requires further exploration. One has also to consider that both ER isoforms (ERα and ERβ) are actively present in the heart tissue [52] and that ERα exists in two isoforms within the heart cytosol and membranes [52]. How fulvestrant affects both isoforms and how ERβ is affected in the presence of fulvestrant have still not been elucidated. Also, our findings may require further proof of evidence by conducting future investigations to study the direct effects of estradiol and/or fulvestrant on the echocardiographic parameters and mitochondrial membrane potential and oxygen consumption rate which will provide the functional evidence of alterations of mitochondrial functions.

## 5. Conclusion

**Fig 5:**
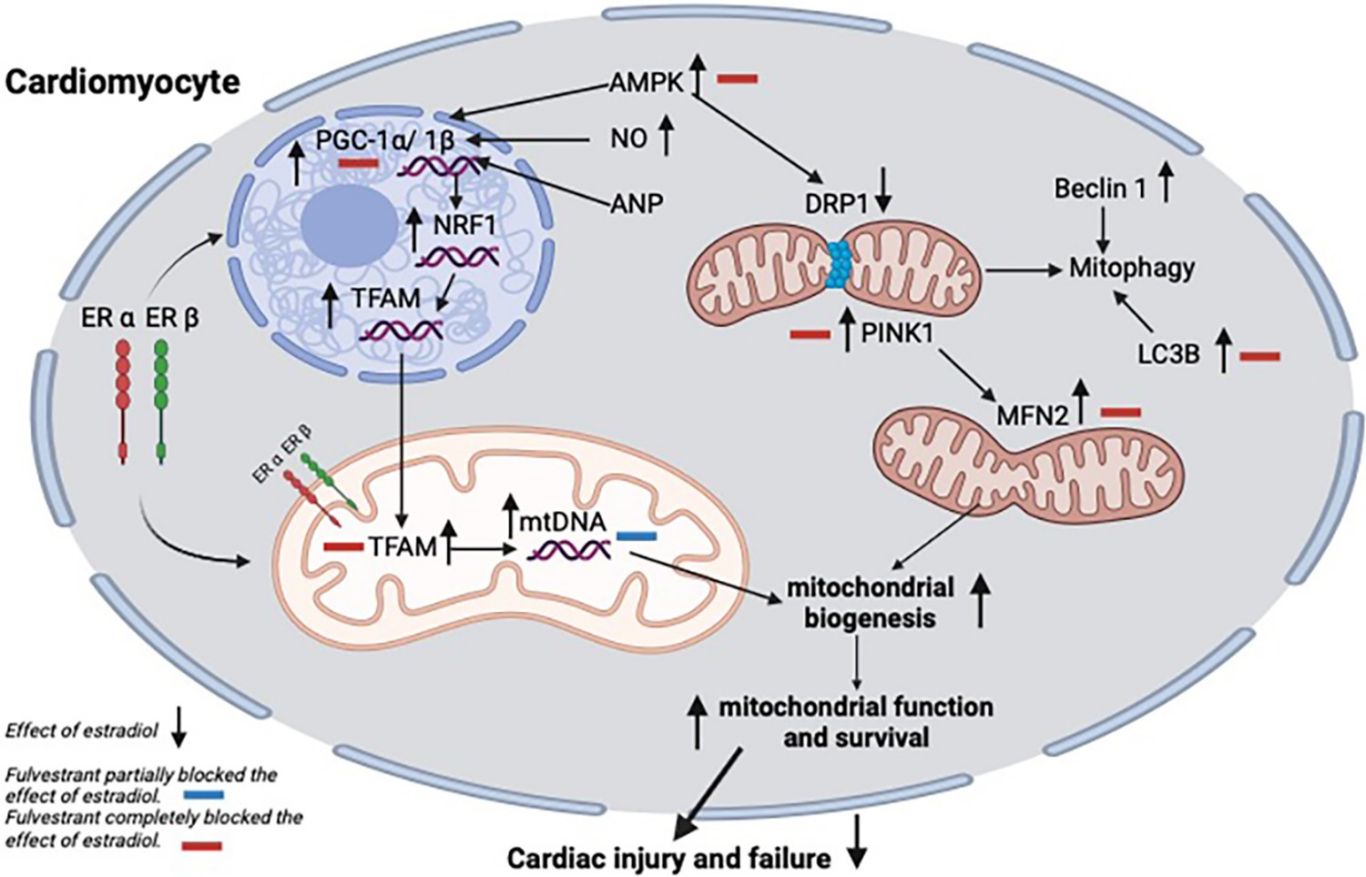
Schematic diagram summarizing the effect of fulvestrant on E2-induced regulation of the cardiac mitochondrial homeostasis in ovariectomized rats. The main targets that have been completely (red) or partially (blue) blocked by fulvestrant are indicated by minus signs. Microtubule-associated protein 1 light chain 3 beta (LC3B), nuclear respiratory factor 1 (NRF1), peroxisome proliferator-activated receptor gamma coactivator 1 (PGC-1), mitochondrial transcription factor A (TFAM), mitochondrial DNA copy number (mtDNA-CN), PTEN-induced putative kinase 1 (PINK1), mitochondrial fusion protein 2 (MFN2), dynamin-related protein 1 (DRP1) and estrogen receptor (ER) alpha (ERα) and beta (ERβ).

The estrogen replacement therapy in the current study had cardioprotective effects. The present study puts AMPK at the core of receptor-mediated E2 actions. The crosstalk of those pathways is illustrated in **Fig 5**, highlighting the main targets that have been completely or partially blocked by fulvestrant indicating their valuable contribution to the E2-related control of myocardial mitochondrial function. This outcome allows a deeper understanding of the role of E2 and ERs in controlling heart function, which is needed to develop new drugs that target cardiovascular diseases in postmenopausal women.

### 6. Future perspectives

As confirmed by fulvestrant, the E2 cardioprotective effects are mainly ER-mediated with the existence of a post-translational control mechanism or the involvement of an alternate estrogenic pathway other than ER in controlling the postmenopausal cardiac mitochondrial energy production machinery that needs further investigation.
